# Pre-Dialysis Systolic Blood Pressure-Variability Is Independently Associated with All-Cause Mortality in Incident Haemodialysis Patients

**DOI:** 10.1371/journal.pone.0086514

**Published:** 2014-01-28

**Authors:** Viknesh Selvarajah, Laura Pasea, Sanjay Ojha, Ian B. Wilkinson, Laurie A. Tomlinson

**Affiliations:** 1 Clinical Pharmacology Unit, Department of Medicine, University of Cambridge, Cambridge, United Kingdom; 2 Department of Nephrology, Cambridge University Hospitals NHS Foundation Trust, Cambridge, United Kingdom; 3 Centre for Applied Medical Statistics, Department of Public Health and Primary Care, Institute of Public Health, University of Cambridge, Cambridge, United Kingdom; 4 Cambridge Clinical Trials Unit, University of Cambridge, Cambridge, United Kingdom; Medical University Innsbruck, Austria

## Abstract

Systolic blood pressure variability is an independent risk factor for mortality and cardiovascular events. Standard measures of blood pressure predict outcome poorly in haemodialysis patients. We investigated whether systolic blood pressure variability was associated with mortality in incident haemodialysis patients. We performed a longitudinal observational study of patients commencing haemodialysis between 2005 and 2011 in East Anglia, UK, excluding patients with cardiovascular events within 6 months of starting haemodialysis. The main exposure was variability independent of the mean (VIM) of systolic blood pressure from short-gap, pre-dialysis blood pressure readings between 3 and 6 months after commencing haemodialysis, and the outcome was all-cause mortality. Of 203 patients, 37 (18.2%) patients died during a mean follow-up of 2.0 (SD 1.3) years. The age and sex-adjusted hazard ratio (HR) for mortality was 1.09 (95% confidence interval (CI) 1.02–1.17) for a one-unit increase of VIM. This was not altered by adjustment for diabetes, prior cardiovascular disease and mean systolic blood pressure (HR 1.09, 95% CI 1.02–1.16). Patients with VIM of systolic blood pressure above the median were 2.4 (95% CI 1.17–4.74) times more likely to die during follow-up than those below the median. Results were similar for all measures of blood pressure variability and further adjustment for type of dialysis access, use of antihypertensives and absolute or variability of fluid intake did not alter these findings. Diastolic blood pressure variability showed no association with all cause mortality. Our study shows that variability of systolic blood pressure is a strong and independent predictor of all-cause mortality in incident haemodialysis patients. Further research is needed to understand the mechanism as this may form a therapeutic target or focus for management.

## Introduction

Mortality is high among patients undergoing haemodialysis treatment for end-stage renal disease (ESRD), and cardiovascular disease is the main cause of death [1]. Hypertension is common in ESRD but, unlike in the general population, is not linearly associated with adverse outcomes [2].

Blood pressure (BP) variability is more closely associated with adverse outcomes in patients with or at risk of vascular disease than ‘usual’ BP [3] and may play a causal role in the progression of organ damage and in triggering vascular events [4]. BP variability is known to be increased in patients with ESRD [5], [6]. Among patients undergoing haemodialysis potential causes of high BP variability such as baroreceptor dysfunction, aortic stiffness and variations in intravascular volume, as well as plausible outcomes such as cerebral small-vessel disease, cerebral haemorrhage and cardiac sudden death are increased compared to the general population [7], [8]. Therefore increased BP variability could provide a strong potential explanation for the increased cardiovascular morbidity and mortality among patients undergoing haemodialysis.

BP variability can be quantified over both the short term, using 24-hour ambulatory BP measurements, and long-term using visit-to-visit BP readings [9]. The optimum method for evaluating BP variability for patients with ESRD is unclear. Previous studies have suggested that visit-to-visit pre-dialysis BP variability is associated with mortality among haemodialysis patients [6], [10], [11]. However, these studies are limited by lack of power and short duration of follow-up, [10], [11] inclusion of prevalent haemodialysis patients [6], [10] and use of measures of blood pressure variability that are associated with average blood pressure levels [10]. Therefore, we planned to investigate whether visit-to-visit pre-dialysis blood pressure variability was associated with mortality among a cohort of patients commencing incident haemodialysis, independently of confounders including average blood pressure.

## Subjects and Methods

### Study Design

We performed a longitudinal cohort study of patients who commenced maintenance haemodialysis between January 1^st^ 2005 and December 12^th^ 2010. Patients were from Addenbrookes Hospital and four satellite units in East Anglia, England. Each underwent haemodialysis three times a week and each dialysis session was at the same time of day and lasted for 3–4 hours.

### Inclusion & Exclusion Criteria

We included all patients who were aged over 18 years at the start of dialysis, had no history of prior renal replacement therapy including transplantation, and remained on dialysis for more than 180 days with no recovery of kidney function. To avoid reverse causality (where underlying cardiovascular disease causes variable blood pressure), we excluded patients who died or suffered cardiovascular events (stroke, transient ischaemic attack (TIA), myocardial infarction, angina or heart failure) in the first 180 days after starting haemodialysis.

### Exposure: Blood Pressure Measurements

BP was measured was measured at the beginning and end of each dialysis session in a seated position by trained dialysis nurses in accordance with routine unit practice and entered immediately into an electronic database. BP was measured using validated oscillometric BP monitor equipped haemodialysis machines (Fresenius 4008S or Nikisso DBB-05), which were maintained as per dialysis unit protocols. For each patient we analysed pre-dialysis BP readings obtained over a consecutive 3-month period, from the 4^th^ to 6^th^ months after commencing haemodialysis. BP readings from the first 3 months after commencing dialysis were not used because this period is associated with acute illness and frequent adjustments to fluid weight and medications, which may have led to overestimation of BP variability in more unwell patients. We used pre-dialysis BP readings to minimise the effect of change in BP due to fluid removal during haemodialysis. We excluded BP readings taken on the first dialysis session of each week to avoid the possible confounding effects of fluid gains over the extended three-day weekend break. Therefore over the 3-month observation period all patients included in the analysis had almost identical numbers of BP readings included in the measurement of the exposure and these were 2 BP readings per week taken 2 days apart on the same day and same time of day for each individual.

### Exposure: Blood Pressure Variability

We measured intra-individual BP variability for both pre-dialysis systolic and diastolic BP using standard deviation, coefficient of variation (standard deviation/mean) and variation independent of the mean (VIM) as previously described [3]. VIM is a transformation of the standard deviation that is uncorrelated with mean BP and is calculated as follows:
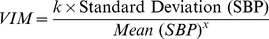



Where *x* is calculated from fitting a power model:
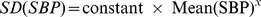



And 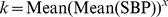.

(i.e. the mean of all SBP readings across all patients to the power of *x*).

For this study, *x* = 0.789 and *k* = 50.552.

### Outcome: All-cause Mortality

The outcome was all-cause mortality during the study period, i.e. from 6 months after the commencement of haemodialysis (at the end of the 3–6 month measurement of the exposure) until the end of follow-up (May 12^th^ 2011). Follow-up was censored if patients received a kidney transplant, switched to peritoneal dialysis or transferred to a different renal unit.

Demographic data, comorbidities and details and dates of censoring events were obtained from the regional renal electronic database and verified using local medical records. Data on medications, interdialytic weight changes, intradialytic weight changes and type of dialysis access were also available. Previous cardiovascular disease (including clinically evident: stroke, transient ischaemic attack (TIA), myocardial infarction, angina, heart failure or peripheral vascular disease) and diabetes were defined as present if recorded in the medical notes. Data on cause of death was not available so it was not possible to analyse cardiovascular mortality separately.

### Statistical Analysis

The definitions of the exposure (including all BP measurements that contributed to the measurement of the exposure and outcome) were defined a priori. The demographic characteristics of groups above and below the median of VIM were compared using Mann-Whitney U tests for continuous variables and chi-squared tests for categorical variables.

We used Cox proportional hazards models to obtain hazard ratios (HR) and 95% confidence intervals (95% CI) for mortality for people with intraindividual visit-to-visit BP variability as a continuous covariate, and also as a categorical covariate, with BP variability dichotomized at the median. We examined whether the proportional hazards assumption was met using partial residual plots for continuous covariates and log (-log) plots for categorical covariates. Kaplan-Meier plots and log-rank tests were also used to compare survival between the two BP variability groups. We carried out univariate analyses of the effect of intraindividual visit-to-visit blood pressure variability on all-cause mortality. We present results adjusted for age and sex as these were considered important a priori confounders. Further covariates examined in multivariate analysis were based on potential evidence of association with mortality among haemodialysis patients; mean systolic BP (SBP), previous cardiovascular events and diabetes. This dataset was complete for all patients. Data on ethnicity was not available but in this region more than 90% of patients are of Caucasian ethnicity. In further separate analyses we adjusted for type of dialysis access (arteriovenous fistula vs indwelling venous catheter), the use of antihypertensive medication (yes vs no) and the number of antihypertensives taken at three months after start of haemodialysis (start of BP variability measurement), the mean intradialytic weight change and the standard deviation of weight change between dialysis sessions as a marker of variability of fluid intake. For this analysis the standard deviation of interdialytic weight change was log transformed due to skewness. We performed the analysis using SPSS version 21.0 (IBM Corp., Armonk, NY).

### Ethics Statement

The study received approval from the Hertfordshire Research Ethics Committee (REC 11/H0311/3). Data was anonymised at source so individual patient consent was not required.

### Data Availability

We are able to provide anonymized data to researchers upon request.

## Results

### Patient Demographics

During the study period 219 patients who were eligible for inclusion commenced maintenance haemodialysis in this region with a total follow up time of 409.4 person years. Of these, we did not include in the analysis 7 patients who died or recovered kidney function and 9 who had cardiovascular events within the first 180 days of haemodialysis. Therefore 203 patients were included in the analysis. From a mean of 25 dialysis sessions, the mean number of BP readings used for the analyses was 25 (SD 1.63). Over a mean follow-up period of 2.0 (SD 1.3) years, 37 patients (18.2%) died. Follow-up was censored for 24 (11.8%) patients who underwent kidney transplantation and 5 (2.5%) who switched to peritoneal dialysis. No patients changed renal units during the follow-up period.

Baseline characteristics of the study population and patients above and below the median VIM of intraindividual visit-to-visit BP variability are shown in Table 1. Patients with VIM of BP variability above the median were more likely to have diabetes but otherwise there were no significant difference between the groups.

**Table 1:** Demographic characteristics of whole study population and groups above and below the median of blood pressure variability.

	Whole group	VIM below or equal to median		VIM above median	*P*-value*
	n = 203	n = 100		n = 103	
**Age (yrs)**	66±15	65±17		68±13	0.55
**Male**	133 (66)	68 (68)		65 (63)	0.46
**Diabetes**	61 (30)	20 (20)		41 (40)	0.002
**Cardiovascular disease**	68 (33)	28 (28)		39 (38)	0.14
**Number of SBP readings**	23.9±2.6	24.0±2.9		23.8±2.3	0.59
**Mean SBP (mmHg)**	144±16	145±16		144±17	0.78
**Mean DBP (mmHg)**	75±10	75±11		74±9	0.31
**Blood pressure medications at 3 months:**					
** ACE-I or ARB**	69 (34)	31 (31)		38 (37)	0.61
** CCB**	92 (45)	44 (44)		48(47)	0.90
** β-blocker**	71 (35)	31 (31)		40 (39)	0.43
** Other**	109 (54)	46 (46)		63 (61)	0.03
** Mean number of agents**	2.0±1.3	1.9±1.3		2.2±1.3	0.17
**Dialysis access at 3 months:**					
**Arteriovenous fistula**	88 (43)	44 (44)		44 (43)	0.85
**Venous catheter**	115 (57)	56 (56)		59 (57)	
**Interdialytic weight gain (kg)**	1.10±0.97	1.04±1.06		1.17±0.87	0.05

Values are expressed as mean ± SD or n (%). ACE-I - Angiotensin Converting Enzyme Inhibitor; ARB - Angiotensin receptor blocker; CCB –Calcium channel blocker, Other – Alpha-blocker, Nitrate, Diuretic, Aldosterone antagonist.

* Mann-Whitney U tests and chi-squared tests were used to compute p-values for continuous and categorical variables respectively.

### Intraindividual Visit-to-visit Systolic Blood Pressure Variability

#### Standard deviation

At an individual level, the mean SD of SBP was 17.4 mmHg (SD 5.1 mmHg). After adjusting for age and sex, the HR for total mortality was 1.07 (95% CI 1.01, 1.13) for every mmHg increase in SD of SBP. This was not attenuated after further adjustment for mean SBP, previous cardiovascular events and diabetes (HR 1.08, 95% CI 1.01, 1.16) (Table 2). After adjusting for age, gender, mean SBP, previous cardiovascular events and diabetes, patients with SD of SBP above the median had a 48% increased risk of death during follow-up but this was not statistically significant (HR 1.48, 95% CI 0.75, 2.91).

**Table 2:** Hazard ratios for one unit increase in measures of systolic BP variability and other covariates from fully adjusted model.

	SD	COV	VIM
	Hazard Ratio	Hazard Ratio	Hazard Ratio
	(95% CI)	(95% CI)	(95% CI)
**BP Variability**	1.08	1.13	1.09
	(1.01, 1.16)	(1.02, 1.24)	(1.02, 1.16)
**Age**	1.03	1.03	1.03
	(0.99, 1.06)	(0.99, 1.06)	(0.99, 1.06)
**Sex**	1.36	1.35	1.35
	(0.65,2.83)	(0.65,2.81)	(0.65,2.82)
**Cardiovascular disease**	0.91	0.92	0.92
	(0.44,1.90)	(0.44, 1.93)	(0.44, 1.92)
**Diabetes**	1.33	1.33	1.33
	(0.63,2.81)	(0.63,2.82)	(0.63,2.82)
**Mean SBP**	0.99	0.99	0.99
	(0.97,1.01)	(0.98, 1.02)	(0.98, 1.02)

SD – standard deviation; COV – coefficient of variation; VIM – Variation independent of the mean; SBP – Systolic blood pressure.

#### Coefficient of variation

For the purpose of regression modelling the coefficient of variation (COV) was multiplied by 100 for each patient to allow sensible interpretation of the hazard ratio. The HR for total mortality, adjusted for age and gender, was 1.13 (95% CI 1.02, 1.24) for every 0.01 increase in COV. After adjusting for age, sex, previous cardiovascular disease, diabetes and mean SBP the HR for total mortality was 1.13 (95% CI 1.02, 1.24) for every 0.01 increase in COV (table 2). After full adjustment, patients with COV of SBP above the median had more than double the risk of death during follow-up (HR 2.08, 95%CI 1.04, 4.16).

#### Variation independent of the mean

**Figure 1:**
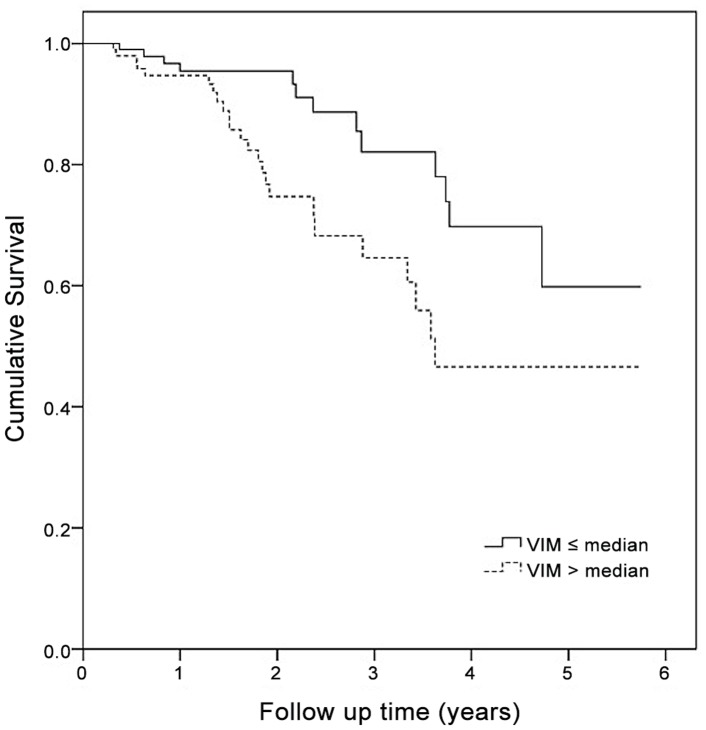
Mortality for patients with high versus low variability of systolic blood pressure.

After adjusting for age and sex, the HR for total mortality was 1.09 (95% CI 1.02, 1.16) for a one-unit increase of VIM. Again, this was not attenuated in the fully adjusted model (HR 1.09, 95% CI 1.02, 1.16). Patients with VIM of SBP above the median were 2.4 (95% CI 1.17, 4.74) times more likely to die during follow-up than those below or equal to the median after adjustment for diabetes, prior cardiovascular disease, gender, age and mean SBP (figure 1).

### Further Analyses

The relationship between VIM of systolic BP and all-cause mortality was not affected by adjustment for the type of dialysis access, the use of antihypertensives, number of antihypertensives used or mean weight change during dialysis sessions (tables S1–S4). In an analysis including the variability of weight change between dialysis sessions (likely to reflect variability of fluid intake) this parameter is also associated with all-cause mortality but does not attenuate the relationship between BP variability and mortality (table S5).

Inclusion of mean diastolic BP in the fully adjusted models made no important difference to the association between any measure of intraindividual visit-to-visit SBP variability and mortality (data not shown).

#### Intraindividual visit-to-visit diastolic blood pressure variability

Measures of intraindividual visit-to-visit diastolic BP variability were not associated with mortality in univariate or multivariate analyses. The age and sex adjusted HR for a one-unit increase in VIM of DBP is 1.02 [95% CI 0.91, 1.14].

## Discussion

Our study shows that intraindividual visit-to-visit variability of systolic BP is associated with all-cause mortality in incident haemodialysis patients, independently of confounders such as age, cardiovascular disease and diabetes. This is seen across measures of BP variability including VIM, which importantly is not correlated with systolic BP. The association between mortality and intraindividual visit-to-visit BP variability is not explained by the type of dialysis access, the use of antihypertensives, absolute fluid intake or variability in fluid intake.

This study has a number of strengths. Duration of haemodialysis is associated with aortic stiffening and autonomic neuropathy, and thus previous renal replacement therapy may be associated with increased BP variability in prevalent cohorts [12], [13]. Therefore, demonstrating these results in a cohort of only incident haemodialysis patients provides greater evidence that this association may be important. We measured intraindividual visit-to-visit BP variability only after 90 days of dialysis, avoiding the early period which may be complicated by acute illness, changes in medications and unstable fluid balance. We only included measurement of pre-dialysis systolic BP taken after the two-day gap to minimize the potential confounding effect of poor compliance with fluid restriction. In addition, we included readings of BP over a prolonged period with very complete data and analyzed the results using three different measures of BP variability. Reverse causality was minimized by excluding patients who had cardiovascular events during measurement of BP variability. This cohort is reasonably large with over two years of follow-up on average and includes all eligible patients in standard clinical care.

However, there are also important limitations. We were not able to adjust for a number of potential cardiovascular risk factors such as smoking, body mass index and cholesterol, as these data were not available. However, among patients with ESRD, the relationship between classic risk factors and risk of adverse events is often weak or reversed and these data may not have affected our findings [14]. We retrospectively analysed routine clinical measurements of blood pressure where technique was as per routine unit practice and not standardized as part of a clinical study protocol. However, it is not clear that use of clinical blood pressure measurements would have led to systematic misclassification of blood pressure variability. Since random measurement error is likely to bias findings to the null, we may have underestimated the strength of the association between BP variability and mortality. Use of routine measurements in an unselected population also means that these results are likely to be generalizable across the United Kingdom and in other countries with similar dialysis schedules and patient populations. In addition, there is potential for selection bias in our findings as follow up was censored when patients received a kidney transplant or transferred to peritoneal dialysis. These people are likely to be healthier, and may have lower BP variability. However, this represents less than 15% of the initial cohort and there was no other loss-to follow-up. Finally, we were not able to examine cause-specific mortality which might have provided greater etiological insight.

Mortality in haemodialysis patients is markedly higher than in the general population and cardiovascular disease is the main cause of death [1]. Conventional measures of BP are linearly associated with cardiovascular risk in the general population [15]. However, among patients undergoing haemodialysis, many studies demonstrate a non-linear relationship between mean BP and mortality [2]. In this group there is increasing evidence that BP variability may be more closely associated with adverse outcomes than conventional measures of BP. Patients with ESRD have greater visit to visit BP variability compared to the general population [5]. Tozawa et al showed that systolic BP variability, quantified by coefficient of variation, predicted all-cause but not cardiovascular mortality in a cohort of 144 Japanese dialysis patients [10]. However, this study was limited by a small number of deaths during the study period (n = 13). A further study by Rossignol and colleagues examined the role of BP variability in a cohort of 397 haemodialysis patients with left ventricular hypertrophy enrolled in an interventional study [16]. They showed that visit-to-visit systolic and diastolic BP variability was associated with a composite end-point of cardiovascular events during follow-up while baseline SBP and DBP were not. A recent study by Chang et al in a cohort of 1844 haemodialysis patients from the HEMO study showed that visit-to-visit SBP variability, quantified by coefficient of variation and average real variability, predicted all-cause and cardiovascular mortality [6]. All three of these studies were limited by inclusion of prevalent haemodialysis patients. Only one study has demonstrated an association between pre-dialysis systolic and diastolic BP variability and all-cause mortality in an incident dialysis cohort [11]. However this study was limited by an extremely short-follow-up (6 months) meaning that reverse causality was a strong potential explanation of their findings. BP variability during haemodialysis has also been associated with all-cause and cardiovascular mortality, as has changing pre-dialysis systolic and diastolic BP over time [17]–[19]. While examining slightly different hypotheses, it is likely that they reflect similar underlying pathophysiology to studies of pre-dialysis systolic BP variability.

The mechanisms that contribute to increased BP variability in patients with ESRD are complex and poorly understood. They include changes in intravascular volume and vasoactive factors (angiotensin 2, endothelin, nitric oxide), reduced arterial compliance, increased sympathetic innervation and alterations of arterial and cardiopulmonary reflexes [20]. Poor or variable compliance with fluid restriction and antihypertensive therapy could also contribute to BP fluctuations. Whether increased BP variability is causal in cardiovascular events and mortality, or whether it is a marker of vascular disease and autonomic dysfunction, is still subject to debate. Strong arguments have been made for changes in BP as a direct mechanism of event causation in cohorts without CKD [4]. The increased BP variability seen in patients with ESRD as well as the high incidence of outcomes plausibly linked to changes in BP such as dementia due to cerebral small vessel disease, hemorrhagic stroke and sudden cardiac death suggest that changes in BP may directly trigger events among dialysis patients. This is important as the use of antihypertensive drugs associated with reduced BP variability are also associated with reduced incidence of stroke [21]. Evidence for improved outcomes with therapeutic regimes targeted to reduce BP variability and prospective data on the effects of different classes of antihypertensives on BP variability in ESRD are still lacking. However, the growing evidence of a link between BP variability and adverse outcomes among haemodialysis patients raises the hope that future strategies to reduce BP fluctuations may be able to improve outcomes for this vulnerable group of patients.

## Conclusions

In summary, this study shows that in standard care, variability of systolic BP from clinical measurements is a strong and independent predictor of all-cause mortality in incident HD patients, even after adjustments for factors such as age, cardiovascular disease and diabetes. This effect is consistent across different measures of BP variability, including VIM, which avoids the confounding effect of average BP. Further research is needed to understand the mechanism as this may form an important therapeutic target or focus for management in this group.

## Supporting Information

Table S1 Results of fully adjusted model for analysis examining the effect of type of dialysis access on relationship between VIM and mortality.
(DOCX)

Table S2 Results of fully adjusted model for analysis examining the effect of antihypertensive use on relationship between VIM and mortality.
(DOCX)

Table S3 Results of fully adjusted model for analysis examining the effect of number of antihypertensives on relationship between VIM and mortality.
(DOCX)

Table S4 Results of fully adjusted model for analysis examining the effect of mean intradialytic weight change (kg) on relationship between VIM and mortality.
(DOCX)

Table S5 Results of fully adjusted model for analysis examining the effect of interdialytic weight variability on relationship between VIM and mortality.
(DOCX)

## References

[1] Parfrey PS, Foley RN (1999) The Clinical Epidemiology of Cardiac Disease in Chronic Renal Failure. Journal of the American Society of Nephrology 10: 1606–1615. 10405218 10.1681/ASN.V1071606

[2] Chang TI (2011) Systolic blood pressure and mortality in patients on hemodialysis. Curr Hypertens Rep 13: 362–369. 21800233 10.1007/s11906-011-0223-x PMC4126801

[3] Rothwell PM, Howard SC, Dolan E, O'Brien E, Dobson JE, et al (2010) Prognostic significance of visit-to-visit variability, maximum systolic blood pressure, and episodic hypertension. The Lancet 375: 895–905. 10.1016/S0140-6736(10)60308-X 20226988

[4] Rothwell PM (2010) Limitations of the usual blood-pressure hypothesis and importance of variability, instability, and episodic hypertension. The Lancet 375: 938–948. 10.1016/S0140-6736(10)60309-1 20226991

[5] Rohrscheib RM, Myers OB, Servilla KS, Adams CD, DD M, et al (2008) Age-related Blood Pressure Patterns and Blood Pressure Variability among Hemodialysis Patients. Clinical Journal of the American Society of Nephrology 3: 1407–1414. 18701616 10.2215/CJN.00110108 PMC2518800

[6] Chang TI, Flythe JE, Brunelli SM, Muntner P, Greene T, et al.. (2013) Visit-to-visit systolic blood pressure variability and outcomes in hemodialysis. J Hum Hypertens: 1–7. doi:10.1038/jhh.2013.49. 10.1038/jhh.2013.49 PMC3795854 23803593

[7] Lacy P, Carr SJ, O'Brien D, Fentum B, Williams B, et al (2006) Reduced glomerular filtration rate in pre-dialysis non-diabetic chronic kidney disease patients is associated with impaired baroreceptor sensitivity and reduced vascular compliance. Clinical Science 110: 101–108. 16171454 10.1042/CS20050192

[8] Wang AYM, Lam CWK, Chan IHS, Wang M, Lui SF, et al (2010) Sudden Cardiac Death in End-Stage Renal Disease Patients: A 5-Year Prospective Analysis. Hypertension 56: 210–216. 20606110 10.1161/HYPERTENSIONAHA.110.151167

[9] Mallamaci F, Tripepi G (2013) Blood pressure variability in chronic kidney disease patients. Blood Purif 36: 58–62. 23735729 10.1159/000351004

[10] Tozawa M, Iseki K, Yoshi S, Fukiyama K (1999) Blood pressure variability as an adverse prognostic risk factor in end-stage renal disease. Nephrology Dialysis Transplantation 14: 1976–1981. 10.1093/ndt/14.8.1976 10462280

[11] Brunelli SM, Thadhani RI, Lynch KE, Ankers ED, Joffe MM, et al (2008) Association between long-term blood pressure variability and mortality among incident hemodialysis patients. Am J Kidney Dis 52: 716–726. 18752875 10.1053/j.ajkd.2008.04.032

[12] Vita G, Bellinghieri G, Trusso A, Costantino G, Santoro D, et al (1999) Uremic autonomic neuropathy studied by spectral analysis of heart rate. Kidney International 56: 232–237. 10411697 10.1046/j.1523-1755.1999.00511.x

[13] Blacher J, Guerin AP, Pannier B, Marchais SJ, London GM (2001) Arterial Calcifications, Arterial Stiffness, and Cardiovascular Risk in End-Stage Renal Disease. Hypertension 38: 938–942. 11641313 10.1161/hy1001.096358

[14] Baigent C, Landray MJ, Wheeler DC (2007) Misleading associations between cholesterol and vascular outcomes in dialysis patients: the need for randomized trials. Seminars in Dialysis 20: 498–503. 17991194 10.1111/j.1525-139X.2007.00340.x

[15] Lewington S, Clarke R, Qizilbash N, Peto R, Collins R, et al (2002) Age-specific relevance of usual blood pressure to vascular mortality: a meta-analysis of individual data for one million adults in 61 prospective studies. Lancet 360: 1903–1913. 12493255 10.1016/s0140-6736(02)11911-8

[16] Rossignol P, Cridlig J, Lehert P, Kessler M, Zannad F (2012) Visit-to-visit blood pressure variability is a strong predictor of cardiovascular events in hemodialysis: insights from FOSIDIAL. Hypertension 60: 339–346. 22777936 10.1161/HYPERTENSIONAHA.111.190397

[17] Flythe JE, Inrig JK, Shafi T, Chang TI, Cape K, et al.. (2013) Association of Intradialytic Blood Pressure Variability With Increased All-Cause and Cardiovascular Mortality in Patients Treated With Long-term Hemodialysis. AJKD: 1–9. 10.1053/j.ajkd.2012.12.023 PMC3660473 23474007

[18] Di Iorio B, Di Micco L, Torraca S, Sirico ML, Guastaferro P, et al (2013) Variability of blood pressure in dialysis patients: a new marker of cardiovascular risk. J Nephrol 26: 173–182. 22419232 10.5301/jn.5000108

[19] Raimann JG, Usvyat LA, Thijssen S, Kotanko P, Rogus J, et al (2012) Blood pressure stability in hemodialysis patients confers a survival advantage: results from a large retrospective cohort study. Kidney International 81: 548–558. 22217879 10.1038/ki.2011.426

[20] Parati G, Ochoa JE, Bilo G (2012) Blood pressure variability, cardiovascular risk, and risk for renal disease progression. Curr Hypertens Rep 14: 421–431. 22903810 10.1007/s11906-012-0290-7

[21] Webb AJS, Fischer U, Mehta Z, Rothwell PM (2010) Effects of antihypertensive-drug class on interindividual variation in blood pressure and risk of stroke: a systematic review and meta-analysis. Lancet 375: 906–915. 20226989 10.1016/S0140-6736(10)60235-8

