# Supplementary material for: Pre-Dialysis Systolic Blood Pressure-Variability Is Independently Associated with All-Cause Mortality in Incident Haemodialysis Patients
Source: PLoS One. 2014 Jan 28;9(1):e86514. doi: 10.1371/journal.pone.0086514 (PMC3904871; doi:10.1371/journal.pone.0086514)
Supplement: Table S2 — Results of fully adjusted model for analysis examining the effect of antihypertensive use on relationship between VIM and mortality. (DOCX) [file pone.0086514.s002.docx]

Supplementary table 2. Results of fully adjusted model for analysis examining the effect of antihypertensive use on relationship between VIM and mortality

| **Covariate** | **Hazard Ratio** | **95% CI** |
| --- | --- | --- |
| **VIM (above vs below median)** | 2.23 | 1.09, 4.56 |
| **Age** | 1.03 | 1.00, 1.06 |
| **Sex** | 1.36 | 0.65, 2.84 |
| **Cardiovascular disease** | 0.98 | 0.46, 2.08 |
| **Diabetes** | 1.32 | 0.63, 2.79 |
| **Mean SBP** | 1.00 | 0.98, 1.02 |
| **Antihypertensives (yes/no)** | 0.93 | 0.36, 2.40 |
