# Supplementary material for: Pre-Dialysis Systolic Blood Pressure-Variability Is Independently Associated with All-Cause Mortality in Incident Haemodialysis Patients
Source: PLoS One. 2014 Jan 28;9(1):e86514. doi: 10.1371/journal.pone.0086514 (PMC3904871; doi:10.1371/journal.pone.0086514)
Supplement: Table S5 — Results of fully adjusted model for analysis examining the effect of interdialytic weight variability on relationship between VIM and mortality. (DOCX) [file pone.0086514.s005.docx]

Supplementary table 5. Results of fully adjusted model for analysis examining the effect of interdialytic weight variability on relationship between VIM and mortality

| **Covariate** | **Hazard Ratio** | **95% CI** |
| --- | --- | --- |
| **VIM (above vs below median)** | 2.20 | 1.08, 4.46 |
| **Age** | 1.03 | 1.00, 1.06 |
| **Sex** | 1.22 | 0.57, 2.60 |
| **Cardiovascular disease** | 1.04 | 0.50, 2.17 |
| **Diabetes** | 1.25 | 0.58, 2.69 |
| **Mean SBP** | 0.99 | 0.98, 1.02 |
| **log (SD of interdialytic weight gain)** | 1.88 | 1.19, 2.97 |
